# Animal emotion: Descriptive and prescriptive definitions and their implications for a comparative perspective

**DOI:** 10.1016/j.applanim.2018.01.008

**Published:** 2018-08

**Authors:** Elizabeth S. Paul, Michael T. Mendl

**Affiliations:** School of Veterinary Sciences, University of Bristol, Langford House, Langford, Bristol, BS40 5DU, UK

**Keywords:** Emotion, Animal emotion, Affect, Definition, Animal behaviour, Consciousness

## Abstract

In recent years there has been a growing research interest in the field of animal emotion. But there is still little agreement about whether and how the word “emotion” should be defined for use in the context of non-human species. Here, we make a distinction between descriptive and prescriptive definitions. Descriptive definitions delineate the ways in which the word emotion is used in everyday life. Prescriptive definitions are used to pick out the set of events that scientific theories of emotion purport to explain. Picking out three prescriptive definitions, we show that the different ways in which emotions are defined correspond to processes that are distributed differentially across the animal kingdom. We propose that these definitions provide a useful starting point for investigating the varying emotional capacities of a wide range of animals, providing a basis for a new, comparative science of emotion.

## Highlights

• Two types of emotion definition can be identified: prescriptive and descriptive.
• Whether or not a particular species can be said to have the capacity for emotion depends on the prescriptive definition being used.
• Prescriptive definitions of emotion provide a basis for a new, comparative science of emotion.

## 1 Introduction

Whether and to what extent different animals experience emotions of one kind or another is an important scientific question; it is also one that to date has proven extremely difficult to answer. This is a key problem for the study and practice of animal welfare, as an animal’s capacity to experience suffering is a prerequisite for any welfare concern. But the extent to which different species share facets of the emotional processes identified in humans is also a question for comparative psychology and neuroscience. What are the fundamental features of emotions, and how far into our evolutionary past do they stretch? When emotional disorders occur in people (as in major depression, generalised anxiety disorder, bipolar disorder, etc.) can they be understood in terms of the responses of emotional processing systems that were originally adapted for a more ancient, even pre-mammalian, way of life? And to what extent are the behavioural and neuropharmacological facets of emotions shared between people and the species used as models to study these disorders? When we can answer questions about the similarities and differences between humans and other species in their emotional processing, it may be possible to find out, not just about the emotional lives of different animals and the implications this has for their welfare, but also about the building blocks, limitations and potential sources of disorder of human emotional processes too.

To date, comparative studies of psychological processes have focused most extensively on cognitive capacities such as short and long-term memory, abstract learning and concept formation (e.g. see Olmstead and Kuhlmeier, 2015; Pearce, 2008), but very little on emotional ones. However, interest in the emotions of animals has increased considerably in recent years, and the time is right to start to redress this imbalance (Panksepp, 2011). To do this, we need to be clear about what we mean when we talk about animal “emotions”. Our starting point is the search for a definition of emotion that can be applied to both human and non-human animals alike (e.g. de Waal, 2011; Lang, 2010; LeDoux, 2012). In this paper, we address the often controversial issue of defining emotions in animals by focusing on the distinction between two types of definition; prescriptive definitions and descriptive definitions. First, we offer a descriptive definition which sets out a broad area of interest for comparative emotion research across humans and other species. We then go on to consider in detail three prescriptive definitions of emotion. Each of these contemporary definitions has been designed to delineate the types of processing that should be considered “emotional”, and each has the potential for drawing a different line between species that can and cannot be considered to have the capacity for emotions. No one of these definitions is superior to the others; we argue that they are all valuable in illustrating the layers of processing that comprise human emotional processing, components of which are likely to be present to differing degrees in different species across the animal kingdom.

## 2 Defining emotion in animals

What is an emotion? And how can we make use of our human understanding of the word to talk about and investigate such processes in animals? For many early ethologists and experimental psychologists, emotion was too human and too quintessentially subjective to be incorporated into the scientific investigation of animals. When Tinbergen was constructing his four questions of ethology (Tinbergen, 1963), the discussion and use of emotion terms in the context of animals was already largely taboo, and worries about the legitimacy of discussing emotion within the animal sciences continued for many years thereafter. So, definitions can be problematic and need to be applied to constructs like emotion with caution. Even attempting to define emotion in the human context has caused problems (e.g. see Gendron, 2010; Izard, 2010), and the difficulty is magnified in non-human animals who cannot tell us whether or what they feel. In these cases, notions of the internal or consciously experienced states are essentially inaccessible. Nevertheless, the past decade or so has seen a dramatic increase in interest, particularly within the neurosciences, but also in ethology, in the topic of emotion and how it can be studied translationally in both humans and animals (e.g. Anderson and Adolphs, 2014; de Waal, 2011; Lang, 2010; LeDoux, 2012; Rolls, 2014).

A major and pertinent distinction when attempting to define emotion is the possibility of constructing both descriptive and prescriptive definitions (Widen and Russell, 2010). A descriptive definition is a dictionary-style definition – one that aims to set out, as comprehensively and accurately as possible, the ways in which a word is used by people, in a given language, and in everyday life (e.g. see LeDoux et al., 2016). A prescriptive definition is somewhat different; it is a definition of the concept or construct that is used to pick out the set of events that a scientific theory (in this case of emotion) purports to explain. Here, emotion theorists take key features of what people think of (i.e. descriptively define as) emotions and say “this is what I am talking about when I use the term emotion in my research” (e.g. Izard, 2010).

Both types of definition are useful for thinking about animal emotions. To start by sketching out a realm of interest when considering the notion and implications of “emotion” in the animal context, a descriptive definition of the word as we use it in the human context is invaluable. If we were to launch straight into making a prescriptive definition for the purposes of research, we may produce one that is too far removed from the real-world human phenomenon that we are interested in, or one that is simply too narrow in its focus.

Below, we have constructed a simple example of a descriptive definition of the English term “emotion”:

“An emotion is a multicomponent response (subjective, physiological, neural, cognitive) to the presentation of a stimulus or event.

The conscious, subjective component of an emotion is generally regarded as its central, key feature.

It is always valenced (i.e. either positive or negative; occasionally both).

It can be intense or mild; long lasting or brief.

The exact type of emotion experienced (e.g. sadness, grief, remorse) will depend on the precise nature of the emotive (emotion-producing) event.

An emotive event can be external or internal (i.e. emotions can be generated by imagination and recollection as well as by events occurring in the environment).

An emotive (i.e. emotion-producing) event is usually one that is in some way important to the goals or relevant to the well-being of the individual.

If an emotive event is reliably predicted, that prediction will also often generate an emotional response.”

This definition identifies the many components that make up people’s emotional experiences and acknowledges the centrality of subjective feelings in our everyday use of the term “emotion”. It also acknowledges the varied, fuzzy and complex nature of the emotion construct (Lang, 2010), with words such as “usually”, “occasionally” or “often”. And while not an exact replica of other previous definitions, it shares the features of many (also like many of these, it may come close to, but perhaps not perfectly encapsulate, the meaning of the word, e.g. see Izard, 2010). For example, “Emotions are affectively charged, subjectively experienced states of awareness (LeDoux, p291, in Ekman and Davidson, 1994); ‘Emotion is a complex set of interactions among subjective and objective factors …. which can (a) give rise to affective experiences such as feelings of arousal, pleasure/displeasure; (b) generate cognitive processes such as emotionally relevant perceptual effects, appraisals, labelling processes; (c) activate widespread physiological adjustments to the arousing conditions; and (d) lead to behaviour that is often, but not always, expressive, goal directed and adaptive’ (Kleinginna and Kleinginna, 1981, p355); “Emotions are typically elicited by external events……typical emotion episodes contain a number of components. There are thoughts, bodily changes, action tendencies, modulations of mental processes such as attention, and conscious feelings” (Prinz, 2004, p3).

By defining emotion in the way we have done, we can point to sorts of comparative problems of emotion that interest or concern us. For example, we might want to say “I am interested in whether certain animals experience emotions in a similar way to humans” or “I want to find out whether birds and mammals differ in the range of emotions that they show”. This is a useful shorthand; a way of indicating that we are interested in this domain of research because of our own human experience of it. It is also a convenient way of outlining the structure and scope of the domain that we want to investigate; the term “emotion” circumscribes a domain that is separate from other English language terms such as “motivation”, “sensation”, “perception” and “cognition”. A descriptive definition such as this can help researchers of emotion delineate the issues they propose to explore.

Different sorts of definitions, however, are needed for the actual execution of emotion research. As Barrett (2006) pointed out, emotions defined in this descriptive manner are cultural constructs, not “natural kinds”; that is, they do not necessarily represent basic biological structures or processes. Moreover, we believe that the term “emotion”, as defined descriptively, is simply too human-centred and quintessentially subjective to be applied without ambiguity to non-human animals (see also LeDoux et al., 2016). For research on animal emotion to go forward, it is useful to be able to put to one side (for a time, at least) concerns and debates about whether and in what ways animals experience emotions consciously. To identify, catalogue and study the core structures and processes of emotion, and to study these in animals in particular, we need objective, prescriptive definitions. This is not to say that conscious experiences of emotion are not central to our understanding of emotions or to our concerns regarding whether and to what extent animals can be said to possess emotional capacities. Rather, we propose that by prescriptively defining emotions and emotional processes in such a way that they can be studied objectively in animals is a vital first step on the path towards any future understanding we may achieve of non-humans’ subjective emotional experiences.

## 3 Prescriptive definitions

In recent years a number of researchers have offered definitions explicitly for the purpose of investigating emotion in animals and humans alike (Anderson and Adolphs, 2014; de Waal, 2011; Lang, 2010; LeDoux, 2012; Rolls, 1999, Rolls, 2005, Rolls, 2014). Others have done this implicitly, focusing on particular, objectively identifiable facets of emotional processing in non-human species (e.g. Panksepp, 1982; Berridge and Kringelbach, 2008). These have been prescriptive, not descriptive definitions. Animal emotion theorists have taken key features of human emotions and said “this is what I’m talking about when I use the word emotion in the context of the animals I study”. Below, we will consider three of these definitions in detail, taking them as examples of the ways in which defining emotions prescriptively in different ways can lead to different answers to key comparative questions about the extent and distribution of emotions in the animal kingdom.

### 3.1 Emotional building blocks

Anderson and Adolphs (2014) define emotion very broadly as “an internal CNS state that gives rise to physiological, behavioural, cognitive (& subjective) responses”. The word “subjective” is kept in parenthesis to indicate that they do not expect that all animals will necessarily produce this component. With this definition, they propose the study of “emotion primitives” in animals. They see these as similar to, but not necessarily homologous with, human emotions. That is, they accept that different phyla and different species within those phyla may have evolved very different approaches to dealing with the similar sets of problems that they encounter in the environment: responding to threat and responding to opportunity.

Central to Anderson and Adolphs (2014) definition is the proposal that emotions comprise four key components or building blocks: Valence, Scalability, Persistence and Generalisation. All four of these, they argue, can be seen as key facets of human emotions. They suspect that these building blocks evolved to serve many independent behavioural and cognitive functions, but that over time they have combined in the brain to produce what we humans now regard as emotion states. But these four primitives, they argue, occur in some form in a very wide range of animal species. And those species which show evidence of all four primitives can be said to be said to have a capacity for “emotion” in this broad sense.

Valence is to do with the opposites that humans experience in emotion: joy vs. anger, happiness vs. sadness. At a subjective level, valence is one of the core features of emotions (and what separates emotional feelings from sensations or perceptions) (Russell, 2003; Barrett et al., 2007; Prinz, 2010). Anderson and Adolphs (2014) point out that in many animals, similarly opposing pairs can be evidenced in behaviour. The primary example of this is approach and withdrawal from an object or stimulus; a young foal, lamb or calf will approach its mother for milk, but withdraw from a novel, loud or moving stimulus (e.g. see Schneirla, 1959). A fish will behave similarly when faced with either a tasty reward or a potential punisher (e.g. Ramasay et al., 2015). And even the roundworm, *C. elegans*, will make use of its olfactory senses to either approach or with draw from stimuli (de Bono and Maricq, 2005; Sengupta, 2007). In fact, the approach/withdraw response is part of the repertoire of most if not all motile animals because it is universally adaptive – enabling an organism to escape from potential threats and approach potential opportunities.

Scalability is another key feature of human emotions that Anderson and Adolphs (2014) argue is both central to their own emotion definition and present in many animal species. Humans can report on the intensity of an emotion and also on the degree of physiological activation or arousal that accompanies a particular valenced state (Duffy, 1957; Russell et al., 1989). This scalability, like valence, seems likely to have fairly universal adaptive significance. If a threat is greater or nearer, it is useful to be able to run away faster. If an opportunity is greater, working harder to obtain it is also likely to be worthwhile (Trimmer et al., 2011).

In humans, emotion words map changing levels of intensity of emotional reactions (e.g. anxiety, fear, panic; glad, happy, ecstatic). These words are illustrative of two key aspects of the scalability of emotions: as the intensity of emotional reactions change, human experiences (and emotional/expressive behavioural) shift both quantitatively and qualitatively. Similar, scaled shifts can be seen in the behaviour and physiology of animals behaving in a valenced manner. For example, a rat that has been food deprived and therefore values its food rewards more highly, is likely to lever press more rapidly, and/or sustain this pressing for longer, in order to obtain them (e.g. see Collier et al., 1992; Hodos, 1961). A domestic hen will run more quickly down a runway to obtain a higher quality food reward (Davies et al., 2015). These are quantitative changes in behaviour that can be recorded and used as measures of the intensity of emotional responses. Qualitative changes, in the form of behavioural switches, can also be observed and used as measures of emotional scalability. For example, octopi and squid switch from crypsis behaviour to ink jetting and propulsion when potential threats increase (e.g. see MacGinitie and MacGinitie, 1968) while rats and many other mammals will switch from freezing to flight behaviour as a predator (or predator-like stimulus) approaches (e.g. Bolles and Fanselow, 1980; Blanchard et al., 1998).

Interestingly, Anderson and Adolphs (2014) note that to date there are relatively few examples of response scaling (either qualitative or quantitative shifts) in invertebrates. Nevertheless, examples do exist, such as the behaviour of squid and octopi described above (MacGinitie and MacGinitie, 1968), the varying levels of behavioural arousal/intensity observed in the fruit fly (*D. melanogaster*) (Chen et al., 2002; Van Swinderen and Andretic, 2003; Greenspan et al., 2001), and observations that crickets (*G. bimaculatus*) show some intensity-related behaviour switching in aggressive encounters (Stevenson et al., 2005).

Persistence is pinpointed as another key characteristic of emotion, according to Anderson and Adolphs’ (2014) prescriptive definition.I In many mammals, heart rate, blood pressure, and the levels of a number of stress hormones can remain elevated for many minutes, and sometimes for hours, following exposure to a threat or other stressor. And in humans, the subjective components of states such as anxiety or sadness can persist even longer, for hours, days, weeks or months. In some instances, these sorts of persistent changes might simply reflect extended, stimulus-response driven (e.g. reflex) effects. But there are also cases where repeated, small-scale stimulation events can lead to extended responses. For example, Drosophila exhibit a persistent state of elevated locomotor activity following repeated stimulation by air puffs; the more puffs applied, or the more intense the puffs, the more persistent the resulting locomotor activity (Lebestky et al., 2009).

Animals that show persistent behavioural, physiological and cognitive responses to rewarding and punishing stimuli may be better able to deal with repeated threats or opportunities that occur in their environment. For example, such a capacity for persistence is likely to be particularly useful for species for whom threats and/or opportunities are temporally correlated; that is, when one good or bad event is associated with a heightened probability of other events of the same valence occurring in the near future (e.g. as would be the case when a reward-rich foraging patch is discovered or an area of high predation risk is entered). Anderson and Adolphs (2014) point out that persistence of response is an observed feature of the behaviour of many species, including invertebrates. However, any possible links between the capacity or tendency to show persistence of responding, and the behavioural niche of an individual species, remains to be explored.

The fourth and final “emotion primitive” that Anderson and Adolphs’ (2014) definition of emotion proposes is Generalization. This, they argue, leads on from persistence, to the extent that an emotion state induced by one stimulus can generalise into subsequent, different contexts and thereby influence responses to other (different) stimuli. And like scalability, the capacity for generalization distinguishes emotional responses, as prescriptively defined by them, from reflexive ones. For example, an insect’s response to an aversive stimulus (e.g. a shadow – Card and Dickinson, 2008), may either be a reflexive response to imminent threat, or it might involve a persistent internal state that can generalise to other contexts or affect subsequent behavioural decisions. Cognitive biases are an example of ongoing responses to threat of punishment or prediction of reward that generalise beyond the trigger stimulus or stimuli (e.g. Harding et al., 2004; Paul et al., 2005; Mendl et al., 2010). In cognitive bias paradigms, animals’ responses to ambiguous stimuli, which might predict higher or lower levels of reward, or either reward or punishment, appear to be shifted according to the animals’ prior experience with unrelated rewards and punishers. While mostly studied in mammals (e.g. Burman et al., 2011; Parker et al., 2014; Douglas et al., 2012) such generalised emotional responses have also been found in birds (e.g. Deakin et al., 2016; Brilot et al., 2010), and even insects (Bateson et al., 2011; Perry et al., 2016).

Emotions as prescriptively defined by Anderson and Adolphs (2014) extend into many branches of the animal kingdom’s phylogenetic tree, including some invertebrates such as the much-studied fruit fly (*D. melanogaster*) or the even, perhaps the roundworm (*C. elegans*). At a practical level, the four building blocks they propose are a potentially useful way of splitting up the multiple processes involved in emotions into easily identifiable units that make studies in such a broad discipline more manageable. They also provide the opportunity to investigate the neural processes involved in each of these different aspects of emotional processing. Anderson and Adolphs (2014) provide good evidence that particular insect species demonstrate some fundamental features of emotional processing, prompting further research questions regarding the extent to which these features are analogous to or homologous with those features that occur in human emotional processing.Their identification of these phylogenetically early “emotion primitives” also prompt further questions about the extent to which other species, and particularly other invertebrates, demonstrate these features, as well as the questions of whether all these features necessarily occur together, and whether the presence of emotion primitives can tell us anything about the capacity of such species to suffer or have poor welfare (Sherwin, 2001).

### 3.2 Emotions as states elicited by instrumental reinforcers

In recent years, Rolls, 1999, Rolls, 2005, Rolls, 2014 has been the most prominent advocate of a reinforcement-based, prescriptive definition of emotion for animals. Following on from a long and slowly developing understanding of the strong link between reinforcement theory in animals and the descriptive construct of emotion in humans (e.g. see Thorndike (1911), Watson (1929), Harlow and Stagner (1933), Amsel (1962), Millenson (1967), Weiskrantz (1968), Gray, 1975, Gray, 1981), Rolls has set out a definition of emotions as “states elicited by rewards and punishers, that is, by instrumental reinforcers” (Rolls, 2014, p14). He goes on to clarify what he means by rewards and punishers: a reward is something for which an animal will work and a punisher is something that an animal will work to escape or avoid.

This is a prescriptive definition that is intended for use in studies of humans and animals alike. It comes close to being an operational definition too – one that, through its objective and procedural nature (i.e. not just “this is what I am talking about when I use the word emotion in my research”, but “this is what you need to do to measure an emotion”), can enable directly comparable studies across species. And it provides the added benefit of proposing a gold standard for the level of emotional response that can be measured – work (Rolls, 1999, Rolls, 2005, Rolls, 2014).

Unlike Anderson and Adolph’s emotion primitives, it seems that Rolls anticipates some degree of homology between human and non-human emotional processes, although, like other animal emotion researchers (e.g. LeDoux, 2012), he points out that this homology does not necessarily imply that emotional states, so defined, are experienced consciously in all animals. From a comparative point of view, the reinforcement-based definition of emotion that Rolls, 1999, Rolls, 2005, Rolls, 2014 proposes provides a reasonably distinct boundary between animal species that exhibit evidence of emotional processing in this sense and those that don’t; Rolls is clear that the capacity to show “true” instrumental reinforcement is a pre-requisite for demonstrating emotion according to his definition, and rules out species that only show responses to rewards and punishers that are evolutionarily established responses or reflexes (Miller and Konorski, 1969). For example, he points out that a single celled organism that “works” to obtain a food reward by swimming towards it across a chemical gradient is not acting on the basis of instrumental reinforcement because it is not performing an arbitrary, learned action, but rather a reflexive response to cues predicting nutrition (Rolls, 2014, p47).

The distinction between truly instrumental and more automatic forms of behavioural control is a well-discussed dividing line in comparative and experimental psychology (e.g. Tolman, 1932; Hull, 1943; Spence, 1956; Bolles, 1972). For example, take a choice situation in which an animal needs to “decide” which action it should perform to obtain a food reward. Effectively, the question the animal has to answer is: which action has the highest value? Critically, the value of prospective actions are determined in more than one way (Dolan and Dayan, 2013). Under Pavlovian control, the “value” of the action is determined by the animal’s evolutionary history, and the action will be effected more or less automatically given the presence of a particular set of stimuli (e.g. a proboscis extension in response to sucrose in the honey bee or a fixed-action crumb peck in response to the sight of food in day old chicks). Under instrumental control, the value of an action is learned as result of an individual’s experiences of the outcomes contingent on that action. The classic example of this is the lever press of an experimental rat (Bolles et al., 1980). In Rolls’ definition, when a food pellet is delivered to the rat, an emotion occurs, and this emotion updates the value (i.e. the expected value for future choice situations) of the lever pressing action. So, instrumentally driven behaviour – in which a novel response is reinforced by a reward (or diminished in frequency by a punishment) is, according to Rolls’ definition, the key behavioural indicator of the presence of emotion in an animal. By extension, only species that can demonstrate “true” instrumental learning can qualify for inclusion as animals with the neural capacity for emotion.

The problem for those researchers trying to establish which species or groups of animals do and do not have a capacity for instrumental learning – and therefore “emotion” in the sense of Rolls’ definition – is that in many (perhaps most) situations, a given behavioural response cannot easily be attributed to either instrumental or Pavlovian valuation processes, and may well be the product of both (Rangel et al., 2008). A number of behavioural phenomena, including constraints on learning (e.g Hinde and Stevenson-Hinde, 1973), autoshaping (Brown and Jenkins, 1968) and negative automaintenance (e.g. Williams and Williams, 1969), all illustrate the difficulty of developing paradigms for generating “true” or “pure” instrumental learning. Famously, Breland and Breland (1961), who trained animals to perform quaint and quirky behaviors for entertainment displays and advertisements, noticed that Pavlovian foraging and feeding behaviors often either fragmented or prevented instrumental training in a range of species, including tame racoons. Similarly in autoshaping, animals appear to perform instrumental responses (e.g. a pigeon performing a feeding-like peck at a cue key that predicts the delivery of food), but this behaviour is simply a classically conditioned foraging response towards the cue (key) that predicts food reward. In negative automaintenance paradigms, the set-up is identical to autoshaping paradigms, except that performing a response such as pecking a key light that predicts food, is inversely associated with the food reward.In such studies, where the instrumental value of an action is pitched against its Pavlovian value, a variety of species including pigeons, rats and dogs (Williams and Williams, 1969; O’Connell, 1979; Sheffield, 1965) have been shown to fail to develop instrumental responses in the face of compelling Pavlovian stimuli.

A potential solution to the difficulties involved in trying to differentiate behaviour resulting from “true” or “pure” instrumental learning concerns the generalised tendency of many animals to approach stimuli that predict reward. Any instrumental training that incorporates approach towards the reward dispenser (e.g. approaching a lever for lever press) is likely to be trained easily because it involves a strong Pavlovian component (Jones et al., 2017). Hershberger’s famous looking glass experiment Hershberger (1986) was designed to see whether chicks (four day old domestic hens) could go against their Pavlovian tendencies to approach a food reward dispenser and learn to do the opposite – to walk away from food – in order to obtain reward. The chicks predominantly failed; they were unable to make use of the instrumental contingency to consistently supress their impulses to approach the sight of food. Whether or not adult chickens can perform this task remains an unresolved question.

A number of other studies have, however, found evidence for instrumental conditioning that is relatively uncontaminated by Pavlovian elements, using instrumental paradigms with bidirectional elements. For example, Grindley (1932) rewarded guinea pigs for turning their heads either to the left or the right at the sound of a buzzer. Not only were both of these opposing actions similarly accessible to training (i.e. neither one appeared to be advantaged or disadvantaged by some hidden Pavlovian control), they were also able to be reversed (e.g. left turn replaced by right turn) by a reversal of the instrumental contingency.

The question of which species of animal unambiguously show the capacity for instrumental learning remains an open one. To date, no systematic attempt has been made to catalogue which animals can and cannot be shown to have this ability (see Dickinson and Balleine, 1994, p3). And there are potential problems with such an endeavour. Many different species would need to be tested, with a clear understanding that factors such as the species’ unique behavioural ecology, and the individual’s current stress levels and prior learning experience could play an important part in determining whether such capacities are found (e.g. see Mendl, 1999; Meagher et al., 2015).

It is generally accepted that the most traditional and commonly employed instrumental paradigm, in which rats learn to produce actions such as lever presses or chain-pulls, can be said to be instrumental in nature (Skinner, 1938; Dickinson and Balleine, 1994). Evidence in support of this comes from Bolles et al. (1980), who found that rats could perform bidirectional instrumental learning of lever pressing and chain pulling. And although mice show some differences from rats in the form of their learning (e.g. Frick et al., 2000, Jones et al., 2017), there is no reason to suppose that their learning of lever pressing and other similar behaviors is anything other than instrumental. But there is still a surprising dearth of evidence for many other species. It seems likely that a fairly large range of animals have the capacity for basic instrumental learning, and should therefore qualify, according to Rolls’ definition, as having the capacity for emotion of some kind. But we simply do not yet know whether and which kinds of birds, reptiles, fish, amphibians, or even invertebrates will show unambiguous evidence for this kind of learning, either using the bidirectional paradigm or other, similar procedures.

### 3.3 Emotions as states that mediate goal directed learning

The third prescriptive definition of emotion that can be applied to both human and non-human animals also concerns instrumental behaviour, but focuses on just one facet of this. It considers a process by which shifts in the value of a reward can gain control of the instrumentally learned actions that allow the animal to gain access to (i.e. work for) that reward. It is well known that motivational shifts (e.g. a growing need for water or food as time since these were last consumed increases) can change the amount of work done by an individual (human or non-human) to achieve a reward. Being able to calculate changes in the expected value of rewards is an important capacity for many animals, enabling them to alter their behavioural decisions readily in the light of new circumstances, including those resulting from their own shifting needs (as in hunger and thirst) and also those resulting from changes in the external world (e.g. when a food source that was once valuable becomes contaminated by bacteria and is now harmful). It is therefore not surprising that a number of different processes or mechanisms have evolved to enable animals to re-value resources according to these many and varied changes. Dickinson and Balleine, 1994, Dickinson and Balleine, 2000, Dickinson and Balleine, 2002, Dickinson and Balleine, 2009, Balleine and Dickinson, 1998a, Balleine and Dickinson, 1998b, Balleine and Dickinson, 1998c focus on one particular process by which this re-evaluation can occur, and in so doing provide a new delineation of emotional processing to add to our list. The theory of emotion that they derived from this is Hedonic Interface Theory (HIT; Dickinson and Balleine, 2009); a theory which aims to explain the origins of subjective, consciously experienced emotions.

Although HIT was not originally thought of as a prescriptive definition of emotion, it can readily be thought of as such. If we leave to one side for a moment the question of whether the type of emotional processing being defined in this way is likely to be consciously experienced or not, Dickinson and Balleine, 1994, Dickinson and Balleine, 2000, Dickinson and Balleine, 2002, Dickinson and Balleine, 2009, Balleine and Dickinson, 1998a, Balleine and Dickinson, 1998b, Balleine and Dickinson, 1998c, like Rolls, 2005, Rolls, 2014, consider emotions to be states that are elicited by rewards (or punishments). But they also go one step further by proposing that emotion, in its role as a hedonic interface, is a constituent part of one type of instrumental conditioning in particular: goal directed learning (Dickinson and Balleine, 2009; Dolan and Dayan, 2013).

In habit-based instrumental conditioning, a given action on the part of the animal (e.g. lever press by a rat) establishes an expected value – a likelihood of being performed again in similar circumstances – on the basis of one or more experiences of the outcome of that action (Wood and Rünger, 2016). That is, the animal learns to lever press more when lever presses are followed reliably by a reward, and less when they are followed reliably by non-reward, the omission of reward, or a punishment. Importantly, this type of learning does not need to incorporate any knowledge of what kind of reward or punishment is expected. That is, the animal may find itself performing an instrumentally reinforced action without any understanding of what reward it will lead to.

Goal-directed instrumental conditioning, on the other hand, is more sophisticated, to the extent that the action becomes associated not only with an expected value, but also an expected identity (Dickinson and Balleine, 1994). That is, there is knowledge (in some form – see Dickinson (1980), for discussion of this) that a reward of a particular kind (e.g. food pellets, water, sucrose solution, etc.) is likely to result from the instrumental action. Colwill and Rescorla (1985) and Adams and Dickinson (1981) were the first to produce demonstrations of this kind of learning, using devaluation paradigms. For example, Colwill and Rescorla’s rats learned that pressing a lever would produce one type of food reward (e.g. sucrose solution) and pulling a chain would produce a different type (e.g. food pellets). The animals then went on to have one or other of their rewards devalued by pairing reward delivery with aversion-inducing Lithium Chloride (LiCl) injections. In subsequent tests of their willingness to perform either of the learned instrumental actions (lever press/chain pull), the frequency of the action associated with the devalued reward was reduced significantly more than that of the non-devalued reward, thereby demonstrating that each of the actions were understood by the rats to be associated with their respective outcomes. Another illustration of goal-directed learning comes from devaluation studies in which animals demonstrate reactions resembling surprise when an expected reward suddenly diminishes in quality or value (e.g. Tinkelpaugh, 1928).

Key to Dickinson and Balliene’s interest in goal directed learning, and its relevance to the prescriptive definition of emotion, is a particular detail regarding the process by which goal directed actions change their value. Based in part on ideas originally put forward by Tolman (1949), Balleine and Dickinson (1991), Balleine (1992) hypothesised that when a *novel* reward such as sucrose solution is devalued, either as a result of pairing with LiCl injection, or simply as a result of satiation, rats would not automatically reduce their instrumental responding to obtain them. Instead, they thought that learning might need to take place in the new state: that the animals would first have to sample the reward following devaluation, in order to understand that under these new and different circumstances, the previously rewarding resource had lost much of it value. Similarly, they hypothesised that an increase in the value of a reward (e.g. a food reward following food deprivation) would also need to be experienced in this new state (of hunger) for the goal-directed instrumental value of that reward to be re-drawn.

Effectively, Balleine and Dickinson (1991), Balleine (1992) were proposing that animals would have to go through a process of discrimination learning to make full use of goal-directed instrumental conditioning, and this is what they found; rats appear to need to learn, that the value of a novel food is greater (i.e. produces a more positive response) when an animal is food deprived, or that of a novel drink is greater when the animal is water deprived. In an early study, Balleine (1992) trained non-food deprived rats to lever press for either high-protein food pellets or a polysaccharide solution. They were then tested in extinction, to see how much lever pressing they would perform to access these rewards, but on this occasion one group of rats was food deprived and another was not. Because these rewards were novel to the rats, and therefore none of the animals had experienced their heightened value during food deprivation, no difference in lever pressing rate was seen. But when another group of rats were given pre-exposure to one or other of the novel food rewards under food deprivation, these rats did change their lever pressing behaviour in line with the new valuation of the rewards.

Dickinson and Balleine (2009) proposed that emotions mediate goal directed learning by bringing together an animal’s Pavlovian and goal-directed control systems’ responses to a rewarding (or punishing) stimulus. Their starting point was the observation that when actions that enable an animal to obtain a reward are under Pavlovian control (that is, when they resulted either from automatic, unlearned stimulus-response associations, or classically conditioned stimulus-response associations) changes in motivation for these rewards (e.g. due to food or water deprivation) cause immediate shifts in both eagerness to work for these rewards and choice (e.g. whether to opt for a food or water reward). But in goal-directed learning, no such automatic re-evaluation takes place (see above – Balleine (1992)). They suggest that an evolved capacity for “emotion”, which they suggest is consciously experienced in these cases, brings together these two systems. In essence, their idea is that emotions, prescriptively defined, evolved for the purposes of binding together information from more primitive, affective systems of reward and punishment valuation and more recently evolved and complex cognitive systems allowing for goal directed learning.

Making use of a prescriptive definition of emotion as states elicited by rewards and punishers in animals that have a capacity not just for instrumental conditioning but goal directed conditioning in particular, limits any potential discussion of emotion in animals to a relatively small number of species. That is, while a large number of species, including many mammals and birds are likely to fall into this category, many more from the wider animal kingdom will fall outside of it. Since they were first introduced, the findings by Dickinson, Balleine and others have been widely influential, particularly in the broader fields of affective neuroscience and reinforcement learning (e.g. Dayan and Balleine, 2002). But at a practical level, demonstrating goal directed learning in animals is a complex and demanding task, resulting in a relative dearth of information regarding its spread across the animal kingdom, including among non-mammalian species. Of these, birds, and particularly some bird groups such as the corvids, are excellent candidates for exhibiting these sorts of capacities (e.g. see Clayton et al., 2005), although the revaluation experiments conducted with rats have not so far been replicated in any animals apart from rats.

## 4 Discussion

We acknowledge that what we humans like to call ‘emotion’ is something that is unique to our species (and even our own individual culture) and that many animals have emotional or emotion-like capacities that are very different to our own, based on neural systems of response to and anticipation of rewards and punishments that are deeply specialised for the niches those animals occupy. But we also argue that empirical questions can be asked about the capacities of different animals to ‘do’ emotion according to different prescriptive definitions of the term. By asking these questions of a wide range of animal species, a much clearer picture will begin to emerge regarding the structure and nature of emotional processes in humans and animals alike. This will be important and relevant, not only for our understanding of animals and their welfare, but also for our understanding of human beings, and the behavioural, physiological and neural underpinnings of the emotions and emotional disorders we experience.

Our proposal for a comparative science of emotion does not aim to ignore or diminish variations in emotional processing and function across species, but to recognise and investigate them. It is already well understood that cognitive capacities and specialisms are uniquely adapted to individual species’ environmental and behavioural niches (e.g. see Macphail, 1982), and emotional capacities are likely to be similarly specialised. This is often seen most clearly amongst island species that have had minimal contact with predators for many generations and as a result become fearless in the face of novel threats (e.g. Blumstein, 2002). While such examples could be viewed as a reduction of a previously extant emotional capacity, other differences between species may concern more fundamental and long-lasting adaptations to an animal’s ecology, such as whether it is predominantly a predator or prey, or a grazer or an opportunistic forager (e.g. Trimmer et al., 2011). We expect to see cross-species differences, but also similarities and hierarchies of capacities. This comparative approach is not, however, an easy challenge. Studying emotional or affective capacities in species not previously thought of as ‘emotional’ can be controversial (Mendl et al., 2010, 2011, Mendl and Paul, 2016). The language used is all important in such cases; perhaps in the future it will be possible to both develop and agree technical terminologies which avoid the confusion and conflict which is currently so common in this area (e.g. see LeDoux, 2012).

Another major challenge for a comparative science of emotion is the sheer quantity of research needed. Not finding evidence of a particular emotion primitive (Anderson and Adolphs, 2014) or of a particular type of learning (Rolls, 2014, Dickinson and Balleine, 2009) is not by itself evidence of its absence, in either an individual or a species. This is an issue that also besets comparative cognitive research, and can only be overcome by the gradual accumulation of data, usually from multiple researchers, taking many different methodological approaches. It is also an issue that is counterpointed by another – publication bias in the communication of negative results. To fully understand emotional and emotion-like processes in a wide range of species, both positive and negative findings will need to be considered, even if their implications are weighted differentially (e.g. see Matosin et al., 2014).

So, which animal species can we say have emotions of some kind and which do not? The short answer is that it depends on how we define emotion. If we define emotion descriptively, it is not possible to say much about animal emotions at all – we can simply point towards them as vague possibilities of similar but not identical constructs. If we define it prescriptively, different definitions will give us different sets of ‘emotional animals’. At first glance, this might seem like an empty conclusion – of course the types of animals that we consider to have emotions will depend on definition. But the devil is in the detail. The prescriptive definitions we have outlined here describe different facets of emotions and some of the different levels or layers of complexity that the emotion systems of humans and many other animals exhibit. Emotions as we experience (and culturally construct) them must ultimately be based on “natural kinds” (Barrett, 2006) – biological processes adapted to the everyday demands of survival and reproduction. But they are not just one process; they are many – like the layers of an onion, different but linked emotional processes have evolved across the course of human evolution and the evolution of other species too. We still have much to discover about the many facets of emotions and their roles in behavioural control (e.g. see Dayan and Berridge, 2014, Bach and Dayan, 2017), and as developments are made, the potential for new prescriptive definitions of emotion will arise.

Like other human-centred constructs, such as intelligence, emotional processes are made up of many parts and there will be no simple linear relationship between capacity for emotionand phylogenetic relatedness or complexity (Pearce, 2008). Nevertheless, broad homologies and analogous processes do exist in the emotional structures and capacities of non-human animals, and the in hierarchical levels of emotional processing that these may represent, and these have been the focus of our attention in the present review. For the present, we have chosen set aside the issue of whether and in what ways non-human animals might be conscious of the emotions we can prescriptively define. Most of the researchers we have cited here do the same, although some (notably Dickinson and Balleine, 2009) have speculated about the types of emotional processing that may be inextricably linked with conscious experience. Perhaps, as both comparative emotion research and consciousness research progress into the future (Boly et al., 2013), it will be possible to make even better speculations and inferences regarding the experiences of emotions in animals.

## Conflict of interest

None.
